# ToF-SIMS mediated analysis of human lung tissue reveals increased iron deposition in COPD (GOLD IV) patients

**DOI:** 10.1038/s41598-019-46471-7

**Published:** 2019-07-11

**Authors:** Neda Najafinobar, Shalini Venkatesan, Lena von Sydow, Magnus Klarqvist, Henric Olsson, Xiao-Hong Zhou, Suzanne M. Cloonan, Per Malmberg

**Affiliations:** 1 Medicinal Chemistry, Respiratory, Inflammation and Autoimmunity, IMED Biotech Unit, AstraZeneca, Gothenburg, Sweden; 2 Target & Translational Science, Respiratory, Inflammation and Autoimmunity, IMED Biotech Unit, AstraZeneca, Gothenburg, Sweden; 3 Early Product Development, Pharm Sci, IMED Biotech Unit, AstraZeneca, Gothenburg, Sweden; 4 Division of Pulmonary and Critical Care Medicine, Joan and Sanford I. Weill Department of Medicine, New York City, New York, USA; 5 Department of Chemistry and Chemical Engineering, Chalmers University of Technology, SE-412 96, Gothenburg, Sweden

**Keywords:** Iron, Mass spectrometry, Chronic obstructive pulmonary disease

## Abstract

Chronic obstructive pulmonary disease (COPD) is a debilitating lung disease that is currently the third leading cause of death worldwide. Recent reports have indicated that dysfunctional iron handling in the lungs of COPD patients may be one contributing factor. However, a number of these studies have been limited to the qualitative assessment of iron levels through histochemical staining or to the expression levels of iron-carrier proteins in cells or bronchoalveolar lavage fluid. In this study, we have used time of flight secondary ion mass spectrometry (ToF-SIMS) to visualize and relatively quantify iron accumulation in lung tissue sections of healthy donors versus severe COPD patients. An IONTOF 5 instrument was used to perform the analysis, and further multivariate analysis was used to analyze the data. An orthogonal partial least squares discriminant analysis (OPLS-DA) score plot revealed good separation between the two groups. This separation was primarily attributed to differences in iron content, as well as differences in other chemical signals possibly associated with lipid species. Further, relative quantitative analysis revealed twelve times higher iron levels in lung tissue sections of COPD patients when compared to healthy donors. In addition, iron accumulation observed within the cells was heterogeneously distributed, indicating cellular compartmentalization.

## Introduction

Chronic obstructive pulmonary disease (COPD) is a debilitating lung disease encompassing airway inflammation (chronic bronchitis), destruction of lung tissue (emphysema) and remodeling of the small airways^1^. With increasing global burden, COPD is currently the third leading cause of death worldwide^2,3^. Cigarette smoke exposure remains one of the main etiological factors for COPD^3^. Tobacco smoke contains 440–1150 µg/g of iron, of which 0.06% is transferred to mainstream cigarette smoke. Lung exposure to iron increases upon regular cigarette smoking^2^, potentially as a result of direct supply of iron through cigarette smoke, or as a result of complexation of host iron by oxygen donor ligands found on cigarette smoke particulate matter. Abnormally increased iron deposition has been reported in the lungs of both current and former smokers^3,4^. Furthermore, levels of non-heme iron and of iron-binding proteins including ferritin, lipocalin-2 and lactoferrin, are increased in lung tissue, sputum, BALF, and cells of COPD patients, relative to non-smokers^4–15^. Although cigarette smoking is the major cause of COPD, occupational exposures, including those to dust, fumes and diesel exhaust, have emerged as important additional risk factors for COPD, especially in nonsmokers^16^. Iron has also been shown to accumulate in lung tissue of people exposed to particulate air pollutions^17^, suggesting this may be another source of iron linked to an increased risk of developing COPD.

Although iron is an essential trace element required for many vital biological processes *viz* oxygen transport and energy production, increased free iron could be detrimental due to its potential to generate free radicals^18^. In addition to environmental risk factors such as cigarette smoke, genetic factors have also been reported to influence COPD susceptibility^1^. Genome wide association studies have identified iron responsive element binding protein 2 (IREB2) as an important candidate for a COPD susceptibility gene, suggesting a functional disease link between abnormal iron metabolism and COPD^2,3^. *IREB2* is a gene on human chromosome 15, coding for iron regulatory protein 2 (IRP2), which plays a vital role in maintaining cellular iron homeostasis^2,18^.

According to the Global initiative for chronic Obstructive Lung Disease (GOLD) criteria, COPD patients are classified into GOLD stage I (Mild), Stage II (Moderate), Stage III (Severe) and Stage IV (Very severe) based on the severity of airflow limitation^19^. In this study, we have employed time of flight secondary ion mass spectrometry (ToF-SIMS) to visualize and relatively quantify iron accumulation in human lung tissue sections of COPD (GOLD IV) patients versus (vs.) healthy donors. Previous studies have employed manual methods for iron quantification in diseased vs. healthy human or mouse lung tissue sections^1,7^, which inevitably introduces risks of biased data interpretation and variability between analyses. ToF-SIMS is a versatile technique that has been used in different areas of research, including analysis of metals^20^, polymers^21^ and complex biological samples. Over the recent years this technique has been introduced for biomedical studies on human tissue samples such as liver^22^, kidney^23^, skeletal muscle^24^ and various forms of cancer such as prostate cancer^25^ and breast cancer^26^.

In this technique, a microfocused beam is impinged on discrete points of a sample surface, generating secondary ions that are analyzed to generate a mass spectrum at each pixel point from the sample. When it comes to chemical analysis of biological samples, ToF-SIMS is a powerful technique in comparison to other single methods, as this technique in parallel can provide identification and localization of substances at high spatial resolution (<400 nm) without the need for labelling^27,28^ and matrix application^29^. However, with imaging mass spectrometry it is still challenging to detect a wide range of ion species with sufficient spatial resolution. To overcome this limitation, the delayed extraction mode has been developed as an alternative method. In this mode, the extracted secondary ions from the sample surface are collected with a certain delay after the arrival of primary ions^30^, which makes it possible to record secondary ion images of ion species with very high spatial resolution and a mass resolution of several thousands in a single run.

In the present study, we successfully demonstrate that ToF-SIMS can be employed for visualization and relative quantification of iron in human lung tissue sections from both healthy and diseased donors (COPD (GOLD IV)). Further, upon application of the delayed extraction mode where spatial resolution down to 400 nm is attainable, we can show that the intracellular iron distribution within cells tentatively identified as macrophages, emerging as the major cell type responsible for iron accumulation in lung tissue^7^, is heterogeneous. Thus, ToF-SIMS appears as a powerful tool to image and relatively quantify lung tissue iron overload, allowing clear differentiation between healthy vs. COPD donors based on their lung tissue iron content. In addition, the technique can distinguish other chemical differences such as alterations in lung tissue lipid composition between the two groups.

## Results

### ToF-SIMS imaging reveals a stronger iron signal in COPD (GOLD IV) patients’ lung tissue vs. healthy donors

**Figure 1:**
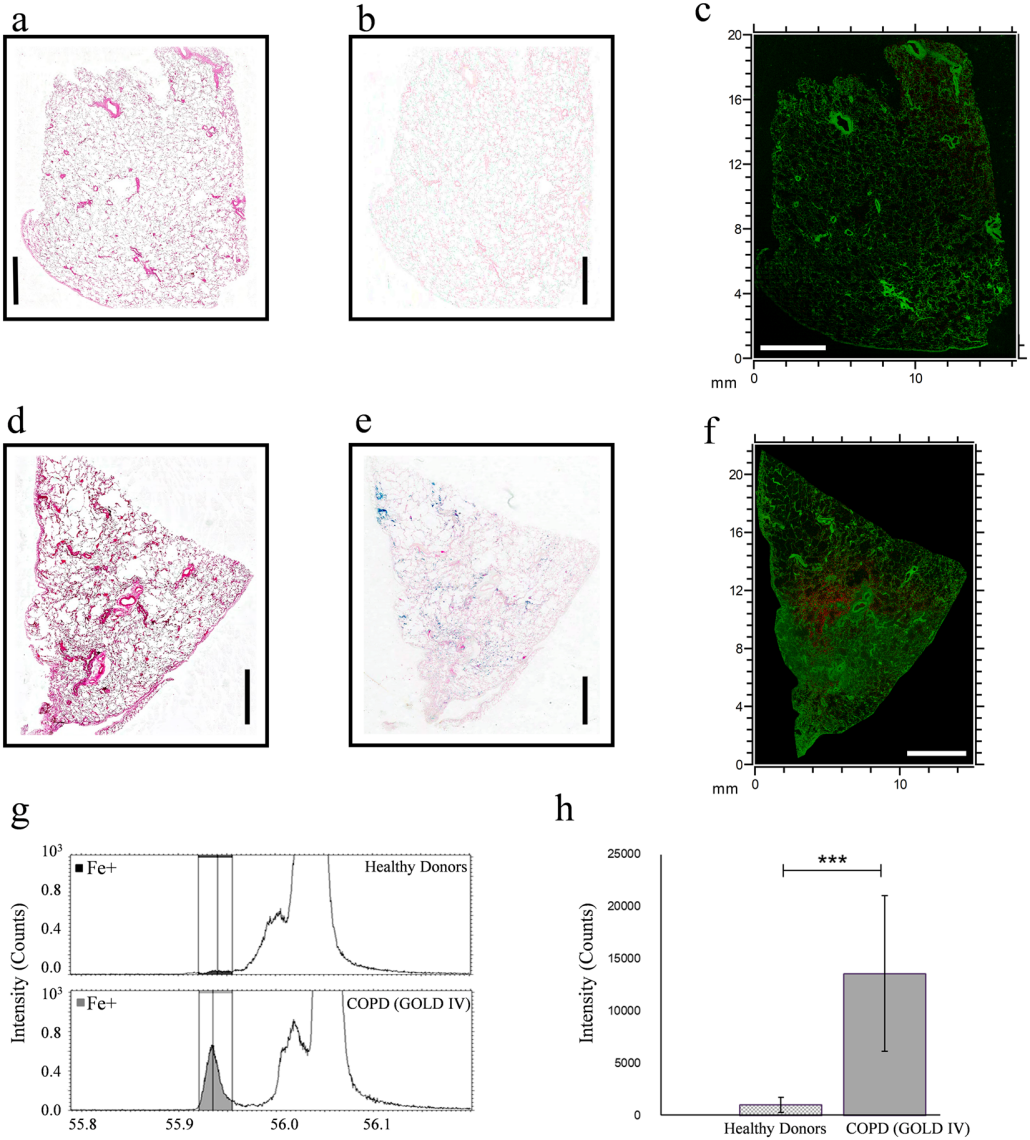
ToF-SIMS imaging reveals stronger iron ion signal in COPD (GOLD IV) patients’ lung tissue vs. healthy donors. The top panel shows representative images of (**a**) H&E staining (**b**) Perls’ Prussian blue (iron) staining, and (**c**) ToF-SIMS overlay image for iron (red, m/z 55.9) and protein fragments (C4H8N+, green, m/z 70.1) in the lung tissue section of a healthy donor across an analysis area of approximately 16 mm x 20 mm. The middle panel (**d**,**e**,**f**) shows the same order of corresponding representative images for a COPD (GOLD IV) patient’s lung tissue section; across an analysis area of approximately 15 mm x 22 mm. Scale bars are 4 mm for all images. The lower panel (g and h) shows ToF-SIMS mediated relative quantification revealing significantly increased iron in COPD (GOLD IV) patients’ lung tissue. (**g**) Normalized intensity for positive species at m/z 55.9 (Fe+), for healthy donors and COPD (GOLD IV) patients’ lung tissue. (**h**) Bar chart showing averaged value of iron intensity in healthy donors and COPD (GOLD IV) patients’ lung tissue. The error bars show 95% confidence level for each peak in 3 tissue sections per group with 5 random areas per tissue. ***p<0.001, using student’s t-test.

In the present study, we employed ToF-SIMS imaging to map the distribution of iron within the complex anatomy of human lung tissue obtained from healthy donors and very severe COPD patients (GOLD IV). H&E staining was performed on healthy donors (Fig. 1a) and COPD patients (Fig. 1d) lung tissue sections to reveal the cellular distribution (pink (cytoplasm) and purple (nucleus) staining) pattern of the tissue sections. Perls’ Prussian (iron) staining performed on the consecutive tissue sections revealed increased iron (blue) staining in COPD (GOLD IV) patient’s lung tissue (Fig. 1e) vs. healthy donors (Fig. 1b). ToF-SIMS macro stage scan images of lung tissue sections analyzed in positive ion mode with Bi_3_^++^ primary ions using the high current bunched mode were used to visualize the total iron signal (red, *m/z* 55.9) showing the signal in contrast to protein fragments (C_4_H_8_N^+^, green, m/z 70.1) in tissue from a healthy donor (Fig. 1c) and a COPD patient (Fig. 1f), respectively. The ion images for iron only for both lung tissue sections are shown in Supplementary Fig. S1. The full scan ToF-SIMS image of a representative healthy donor’s lung tissue showed only a very weak signal for iron (Figs 1c, S1a), whereas the ion signal for iron in lung tissue from a very severe COPD (GOLD IV) patient was significantly stronger (Figs 1f, S1b).

Next, iron ion imaging of a COPD (GOLD IV) patient’s lung tissue was compared to standard histochemical iron (Perls’ Prussian blue) staining performed on a consecutive lung tissue section. Correlations regarding lung tissue regions of iron overloading was observed between the two methods (Fig. 1e,f), however the ToF-SIMS data showed several areas with apparent higher iron signals not detectable with Perls’ Prussian blue. In line with the poor iron ion signal in Fig. 1c, no evident iron staining was observed in a healthy donor’s lung tissue section as seen in Fig. 1b. This validates ToF-SIMS as a powerful technique to localize, map, and visualize the distribution of iron in human lung tissue at a high spatial resolution.

It is worth noting though, that for both conventional histochemical iron staining and ToF-SIMS analysis, the paraffin fixed lung tissue sections were de-paraffinized using xylene. Since xylene is an aggressive chemical, the risk for iron delocalization and leaching needs to be considered. Still, the good correlation between iron staining and ToF-SIMS imaging performed on consecutive serial sections indicates that such delocalization is limited. Another potential issue is that since the sampling area is relatively big (approximately 15*22 mm), charging and topographic effects can be present. Normalisation of the iron signal to the total ion signal did however, not significantly alter the ion image for iron. Also, the quality of the obtained spectra based on achieved mass resolution and lack of peak tailing (Supplementary Fig. S2), indicates that such problems are less pronounced.

### Relative quantification of iron using ToF-SIMS reveals significantly increased iron in COPD (GOLD IV) patients’ lung tissue

Studies so far^1,7^ have mostly relied on manual quantification of iron positive cells by histochemical staining (Perls’ Prussian or Perls’-DAB) of lung tissue sections. In this study, we used ToF-SIMS for relative quantification of iron in lung tissue sections of healthy donors vs. COPD (GOLD IV) patients. For this purpose, three sections from three different donors in each group were chosen, five areas per section were randomly selected for iron imaging, and the ion signal for iron was analyzed and averaged. The relative quantification revealed approximately twelve times higher levels of iron in COPD (GOLD IV) patients’ lung tissue sections compared to that of healthy donors (Fig. 1g,h). These findings are in agreement with a previous study, which showed that cigarette smoking increases iron deposition systematically in alveolar macrophages from human subjects with COPD, and that the percentage of iron positive macrophages were increased with COPD severity^7^.

### Heterogeneous iron distribution at the cellular levels

In continuation to the observation of a strong iron ion signal from the COPD (GOLD IV) patients´ lung tissue, we decided to investigate whether the pattern of iron distribution could be assessed down to the cellular or the subcellular level. To achieve this, we used the ToF-SIMS delayed extraction mode, allowing lateral resolution down to 400 nm while maintaining a high mass resolution. Applying this technique is an efficient way to overcome the issue of compromising between a high mass resolution and a sub-µm lateral resolution in conventional ToF-SIMS.

**Figure 2:**
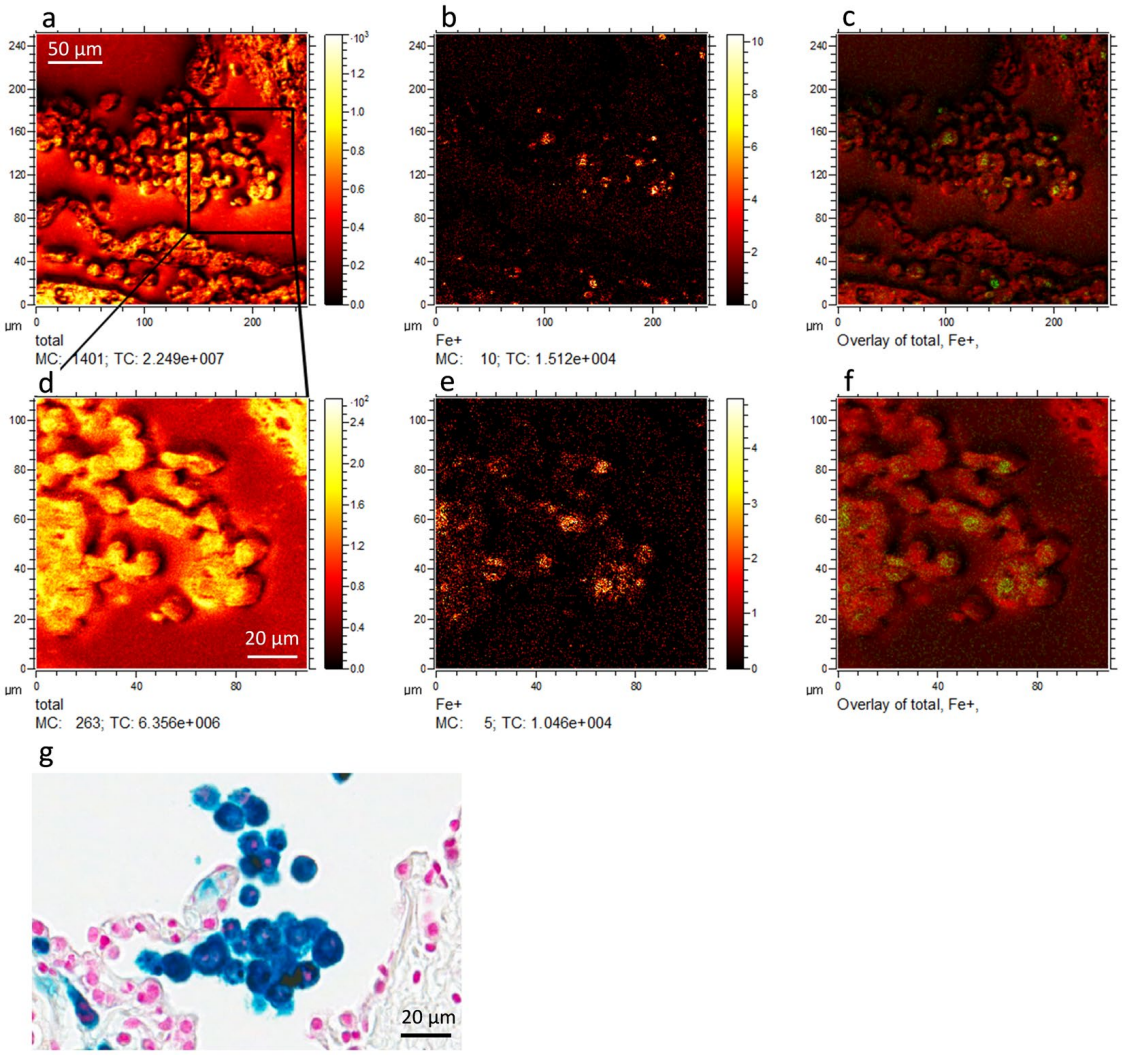
Heterogeneous iron distribution has been shown in iron positive cells of COPD (GOLD IV) patients’ lung tissue. Top panel shows representative (**a**) total ion image, (**b**) the iron ion signal image, and (**c**) the overlay of the two images obtained from COPD (GOLD IV) patients’ lung tissue using delayed extraction mode in ToF-SIMS instrument. Bottom panel shows a region of image (**a**) further magnified and their corresponding (**d**) total ion image, (**e**) iron ion signal image and (**f**) the overlay of both. Figure 2g shows a consecutive section of Perls’ Prussian blue/ nuclear fast red stained COPD (GOLD IV) patient lung tissue including alveolar macrophages (tentatively identified). Scale bar for figure (**a**–**c**) 50µm and figure (**d**–**g**) 20µm.

The heterogeneous nature of iron distribution was revealed by overlaying microscopic total ion and iron signal images (Fig. 2) of COPD (GOLD IV) patient’s lung tissue sections. The iron enriched cell type was tentatively identified as macrophages based on morphology and previously reported data^7^ and by analyzing consecutive sections with Perls’ Prussian blue/ nuclear fast red as seen in Fig. 2g. The punctuate subcellular distribution of iron is in line with the recent observation that cigarette smoke can induce iron loading in mitochondria^1^. Mitochondria are the main consumers of intracellular iron, and iron regulation by mitochondria may play a vital role in many cellular processes^1^. Similarly, lysosomal iron turnover is also an important component of intracellular iron regulation^31^ and the contribution of lysosomal iron to these subcellular regions should also be considered. The exact subcellular localization of iron inside these macrophages remains to be determined and is currently the subject of further investigation.

**Figure 3:**
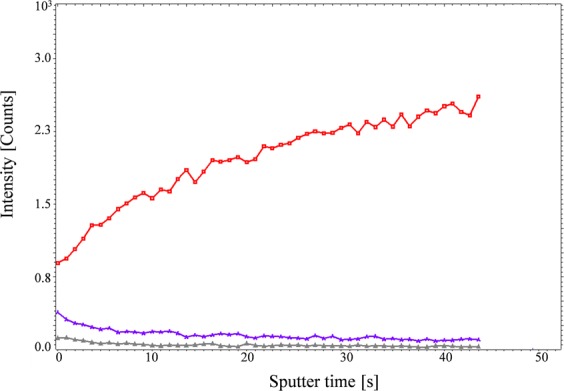
Depth profiling of iron positive cells in COPD (GOLD IV) patients’ lung tissue. Red line indicates the iron signal, whereas the blue and gray lines depict phosphatidyl choline (PC) and cholesterol respectively.

Following the identification of heterogeneous distribution of iron within the iron positive cells, we performed depth profiling on these cells to confirm that the measured iron signal was from intracellular deposits. In this mode, the ion intensity in the z-depth, as sputter time (s), of the tissue section can be monitored. The results clearly demonstrated that when entering the cells, the signal from iron increased whereas the signal from plasma membrane lipids including phosphatidylethanolamine (PE) and phosphatidylcholine (PC) decreased (Fig. 3), indicating that the imaged iron signal emanated only from intracellular iron.

### Data analysis by PCA and OPLS-DA: OPLS-DA shows a much-improved separation between healthy donors and COPD (GOLD IV) compared to PCA

Next, multivariate data analysis was performed to explore whether the lung tissue ToF-SIMS spectra could differentiate and distinguish healthy control subjects from COPD patients based on the chemical composition of the tissue.

**Figure 4:**
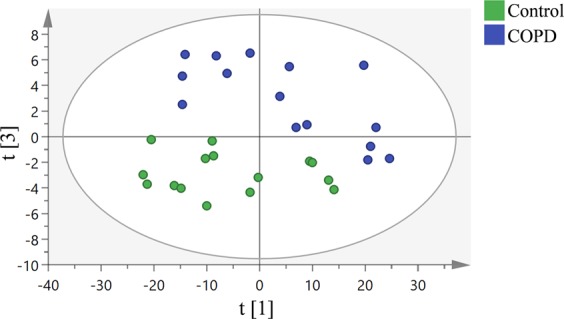
PCA analysis of positive mode data from ToF-SIMS analysis of 3 human lung samples per group (healthy donor and COPD (GOLD IV)) with n = 3 replicates per sample. Score plot of the first principle component (t[1]) vs. the third principle component (t[3]) from the spectra. Green and blue dots represent healthy donors and COPD (GOLD IV) samples respectively.

Principle component analysis (PCA) was carried out on the collected and normalized spectra obtained from ToF-SIMS analysis of three different lung tissue samples from each group (healthy donors and COPD (GOLD IV)) with five regions for analysis randomly chosen on each sample. In the PCA analysis, the two groups could be discerned using the first (PC1: t[1]) vs. the third (PC2: t[3]) principal component (Fig. 4). However, the groups weren’t very well separated as the data was influenced by variations within the groups, resulting in scattered data points. This was an anticipated variation given that the data was collected from fifteen different scanning regions from only three different human samples in each group, which is a low n-number considering the inherent biological variation between donors and samples.

**Figure 5:**
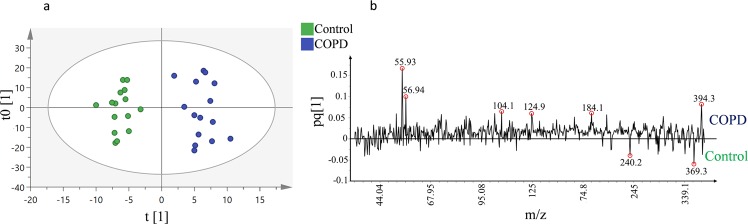
OPLS-DA analysis of positive mode data from the ToF-SIMS analysis of 3 human lung samples per group (healthy donor and COPD (GOLD IV)) with n = 3 replicates per sample. (**a**) Score plot of the ToF-SIMS data showing distinctive separation between healthy donors’ (Green dots) and COPD (GOLD IV) (Blue dots) samples. This model explains 85% of the variation in the data set (R2X cumulative) with predictive power of 0.83% (Q^2^ cumulative). (**b**) Corresponding loading plot representing the m/z peaks with strong impact on the separation between the two groups.

To improve the separation between the groups, we performed OPLS-DA, a variant of OPLS, focusing specially on class separation^32^. With the same dataset, OPLS-DA analysis revealed an excellent separation between healthy donors and COPD (GOLD IV) patients (Fig. 5a). Clearly, the differences between the samples are substantial, allowing them to be chemically differentiated into two distinct groups. Although, the samples for COPD group are from the same stage (GOLD IV) of disease, it is evident that there are still variations remaining among the individuals and within (heterogeneous iron distribution in the lung) giving rise to sample variation.

The loading plot corresponding to the OPLS-DA score plot revealed that iron was one of the most prominent biological masses amongst the highest loading peaks (*m/z* 55.9) and thus a major driver of the separation between the two groups (Fig. 5b). Apart from iron, the peaks with masses of *m/z* 104, 124.9, 184.1, 240.2, 369.3 and 394.3 were differentially expressed (over- or under expressed) in the COPD (GOLD IV) samples. These peaks are likely to be emanating from different lipids, as it has been previously reported that more than 210 lipids were differentially expressed in COPD, including a large portion of sphingolipids that were significantly upregulated in smokers with COPD^33–35^. The lung lipidome and lipid signaling have been implicated in regulating many of the biological processes associated with COPD. Several inflammatory cytokines upregulated in COPD have the potential to modulate the activity of lipid-modifying enzymes such as phospholipases, sphingomyelinases and sphingosine kinases, which might consequently result in an altered lipid composition that could affect cellular integrity. For instance, it has been shown that dysregulation of the sphingolipid metabolism may be an important driver of progressive alveolar destruction and remodeling in COPD^35,36^. Thus, analysis of the COPD lung lipidome may provide novel approaches to biomarker development and identification of potential therapeutic targets^33^. However, this is not in the scope of this paper and further investigation is required to better understand the effect of COPD on lipid expression.

## Discussion

ToF-SIMS is a novel analytical technique gaining increasing importance in life sciences due to its relatively high spatial resolution and sensitivity combined with its scope for deep chemical profiling analysis. Thus, mass spectrometry imaging provides an excellent opportunity to obtain an unprecedented amount of information on the distribution of elements and molecules in biological samples^37^. In the field of pulmonary biology, the ToF-SIMS technique has recently been applied to detect the exact localization of inhaled nanoparticles (*viz* zirconium dioxide (ZrO_2_)^38^ and silicon dioxide (SiO_2_)^39^) in rat lung tissue sections and in aerosolization research to study the migration of excipients onto the surface of inhaled drugs^40–42^. ToF-SIMS has also been employed in lipidomics profiling of lung tissue from *Pseudomonas aeruginosa* infected Cystic Fibrosis Transmembrane Conductance Regulator deficient (CFTR−/−) mice. This study reported a significant increase in cholesteryl sulfate (*m/z* 465.4) in lung epithelium from CFTR−/− mice in response to bacterial infection^43^. ToF-SIMS has also been routinely used in studying the lateral organization of lipids and proteins in pulmonary surfactant systems^44,45^.

Our study has several important strengths. To the best of our knowledge, we are the first to successfully use ToF-SIMS to image iron in human lung tissue sections at a spatial resolution of 400 nm and to perform relative iron quantification. This application of ToF-SIMS is highly valuable as it is more precise in quantification and less error prone compared to conventional histochemical methods. For example, unlike common histochemical methods, ToF-SIMS detection of iron is not altered by anthracotic material (accumulation of carbon in the lungs due to repeated exposure to particles)^46^, nor dependent on histopathologic scoring, which is at best semi-quantitative. Relative quantification of iron load by ToF-SIMS revealed approximately twelve times higher levels of iron in lung tissue sections from severe COPD patients as compared to healthy control donors. This agrees with a previous qualitative assessment showing increased iron deposition in very severe COPD patients’ lung tissue using the Perls’-DAB histochemical iron staining protocol^7^. The same study^7^ identifies alveolar macrophages as the predominant iron positive cell population. Despite clear evidence of iron accumulation in lung tissue obtained from COPD patients, the source of iron remains obscure. Increased iron content in COPD lungs could possibly result from cigarette smoke exposure^2,4^, genetic predisposition *e.g*. IREB2 polymorphism^2,3^, alveolar microhemorrhage^47^, systemic inflammation^47^, air pollutants or other unknown factors. Iron laden alveolar macrophages have also been reported in Idiopathic Pulmonary Fibrosis^47^ and elevated iron levels have been found in the respiratory tracts of cystic fibrosis patients^48^. In addition to respiratory diseases, dysregulated iron metabolism and iron accumulation have been observed in neurodegenerative disease conditions such as Alzheimer’s disease, Parkinson’s disease, Multiple sclerosis and Amyotropic Lateral Sclerosis^49,50^.

Our study also has limitations. ToF-SIMS does not discriminate between oxidized, ferric (Fe^3+^) iron and soluble ferrous (Fe^2+^) iron. At physiological pH, iron pre-dominantly exists as insoluble ferric iron, bound to carrier proteins such as transferrin^51^, ferritin^9^, lactoferrin^52^, ceruloplasmin^53^ and/or lipocalin 2^8^, or unbound in the form of cell free hemoglobin/heme or non-transferrin bound iron (citrate or acetate bound iron), all of which are found in the BALF. To be absorbed, iron must be in the ferrous (Fe^2+^) state. Intracellular Fe^2+^ is then either stored (ferritin, mitochondrial ferritin, lysosome etc.) or utilized for essential biologic processes including heme synthesis and Fe-S cluster synthesis. Under conditions of excessive intracellular iron, surplus iron may be deposited as insoluble polymeric iron(III) with oxo-bridges termed hemosiderin^54^. In this study we are unable to determine if the iron detected by ToF-SIMS is ferric, ferrous, insoluble or soluble or if it is bound to a specific protein. Further studies are required to determine the nature of this accumulated iron in COPD.

Iron is an essential trace element required for various biological processes, but when overabundant it may become pathogenic due to generation of free radicals, oxidative stress, and facilitation of bacterial infections^18,48^. Mitochondria are major cellular organelles that play a key role in iron metabolism, and they harbor major pathways for iron utilization and production of iron containing protein such as iron- sulphur cluster biosynthesis and heme synthesis^55^. Recently, it was demonstrated that mitochondrial iron loading holds a pivotal role in the development of bronchitis and emphysema, and that chelation of mitochondrial iron may alleviate cigarette smoke induced impairment of mucociliary clearance, pulmonary inflammation and lung injury in mice with experimental COPD^1^. Whether the heterogeneous iron distribution pattern observed in this study is due to iron accumulation in selective cellular organelles such as mitochondria or lysosomes needs to be determined in future studies.

## Conclusion

In this study, we demonstrate for the first time that ToF-SIMS can be successfully employed to image iron in human lung tissue sections, and that using delayed extraction mode enables imaging of the iron distribution pattern at cellular levels. Further, we show that relative quantification of lung tissue iron content is feasible with ToF-SIMS and that twelve times higher iron levels are present in lung tissue sections from COPD (GOLD IV) patients as compared to healthy donors. Most of this iron was shown to be heterogeneously distributed within the iron-positive cells, consistent with accumulation within discrete cellular organelles such as mitochondria or lysosomes. Using multivariate analysis OPLS-DA, we show that COPD patients and healthy controls can be clearly separated on basis of the chemical composition of their lung tissue. The iron signal was found to be a major driver causing this separation. However, other species, *e.g*. lipids, may contribute to the observed separation, which needs to be investigated in future studies.

## Methods

### Healthy Donors and COPD (GOLD IV) patients’ information

Transplanted lung tissue from three end stage COPD (GOLD IV) patients and three healthy donors age-matched to the COPD group were employed for this study. The studies were approved by the Swedish Research Ethical Committee (Gothenburg FEK 675-12/2012, Lund FEK 91/2006). All research was performed in accordance with relevant guidelines and regulations. Signed informed consent forms were received from the study subjects or their closest relatives.

### Human lung tissue Sample Preparation

The lung tissue was dissected into small pieces and fixed with 4% buffered formalin over-night, followed by dehydration and paraffin embedding process as routinely performed for biobank storage and described previously*^56,57^. 3 µm thick tissue sections were mounted on microscopic glass slides and used for ToF-SIMS imaging and Perls’ Prussian blue iron staining.

### ToF-SIMS Imaging and data analysis

A ToF-SIMS 5 instrument (IONTOF GmbH, Germany) equipped with a liquid metal primary ion source was used for all ToF-SIMS experiments. Bi_3_^++^ primary ions at 25 keV energy were applied to record ToF-SIMS spectra of positive ions. Both the high current bunched mode with a mass resolution of m/∆m = 6000 fwhm at *m/z* 100, as well as the delayed extraction mode, with a mass resolution of m/∆m = 4000 fwhm at m/z 100 were used. The delayed extraction was set to an extraction voltage of 2 kV, the rise time to 40 ns and the delay was adjusted to 5 µs. The pulsed primary ion current was 0.3 pA and 0.25 pA for high current bunched mode and delayed extraction mode respectively. The maximum ion dose density of Bi_3_^++^ was kept below 4 × 10^11^ cm^−2^. To avoid charging effects, electron flooding was used. In total three sections obtained from three different donors in each group (healthy donor and COPD) with five random areas from each section were analyzed. Depth profile analysis was performed using pulsed Bi_3_^++^ gun (0.26 pA) while sputtering was carried out with a C_60_^++^ beam with a current of 0.78 nA.

The Surface Lab software (version 6.3 ION-ToF, GmbH, Germany) was used to analyze all ToF-SIMS data. For mass calibration, signals of [C]^+^, [CH]^+^, [CH2]^+^, [CH3]^+^, and [C5H15PNO4]^+^ were used. Spectra obtained from individual samples were analyzed using the search peaks function in the Surface Lab software. Parameters employed to select peaks were as followed: counts >100, S/N > 3, width 0.8 Da. In the following step, the complete data set consisting of 804 peaks were normalized to the primary ion dose of the respective spectra and then subjected to principle component analysis (PCA) and orthogonal partial least squares discriminant analysis (OPLS-DA) data analysis using SIMCA (version 13.0, Umetrics, Umea, Sweden). Low mass peaks, *m/z* below 30, were excluded from the analysis as they were related to redundant and not chemically specific peaks such as hydrocarbon clusters. The scores and loading plots revealed which ions (*m/z* values) were responsible for most of the variation between the two groups, healthy and COPD.

### Hematoxylin and Eosin (H&E) staining

Formalin-fixed, paraffin embedded lung tissue sections were deparaffinized and rehydrated using xylene and decreasing concentrations of alcohols and washed in de-ionized water. Tissue sections were then stained with Hematoxylin (HistoLab, Sweden) for 8 minutes and kept under running tap water for 5 minutes to allow stain to develop. The sections were then stained with eosin (Histolab, Sweden) for 5 minutes, after which they were dehydrated through treatment with increasing concentrations of alcohols and xylene. Finally, the tissue sections were mounted and scanned using Aperio CS2 scanner (Leica Biosystems, Germany), and analyzed using Aperio ImageScope (Leica Biosystems, Germany).

### Perls’ Prussian blue/Iron staining

Formalin-fixed, paraffin embedded lung tissue sections were deparaffinized and rehydrated using xylene and decreasing concentrations of alcohols and washed in de-ionized water. Slides were then incubated in Perls’ stain (Iron assay kit, Sigma-Aldrich, Sweden) as per the manufacturer’s protocol for 30 minutes at room temperature (RT) and then washed with distilled water. For nuclear staining, slides were incubated with nuclear fast-red solution and incubated for 5 minutes at RT. Following, the slides were washed with distilled water for 5 minutes, dehydrated and mounted. Slides were scanned using Aperio CS2 scanner (Leica Biosystems, Germany) and analyzed using Aperio ImageScope (Leica Biosystems, Germany).

## Supplementary information

Supplementary information
