# Supplementary material for: ToF-SIMS mediated analysis of human lung tissue reveals increased iron deposition in COPD (GOLD IV) patients
Source: Sci Rep. 2019 Jul 11;9:10060. doi: 10.1038/s41598-019-46471-7 (PMC6624371; doi:10.1038/s41598-019-46471-7)
Supplement: Supplementary file 1 — Supplementary information [file 41598_2019_46471_MOESM1_ESM.docx]

**ToF-SIMS mediated analysis of human lung tissue reveals increased iron deposition in COPD (GOLD IV) patients.**

^1^Neda Najafinobar, ^2^Shalini Venkatesan, ^1^Lena Von Sydow, ^3^Magnus Klarqvist, ^2^Henric Olsson, ^2^Xiao-Hong Zhou, ^4^Suzanne M. Cloonan, ^5^Per Malmberg*

^1^Medicinal Chemistry, Respiratory, Inflammation and Autoimmunity, IMED Biotech Unit, AstraZeneca, Gothenburg, Sweden

^2^Target & Translational Science, Respiratory, Inflammation and Autoimmunity, IMED Biotech Unit, AstraZeneca, Gothenburg, Sweden

^3^Early Product Development, Pharm Sci, IMED Biotech Unit, AstraZeneca, Gothenburg, Sweden

^4^Division of Pulmonary and Critical Care Medicine, Joan and Sanford I. Weill Department of Medicine, New York City, New York, USA.

^5^Department of Chemistry and Chemical Engineering, Chalmers University of Technology, SE-412 96 Gothenburg, Sweden

* malmper@chalmers.se


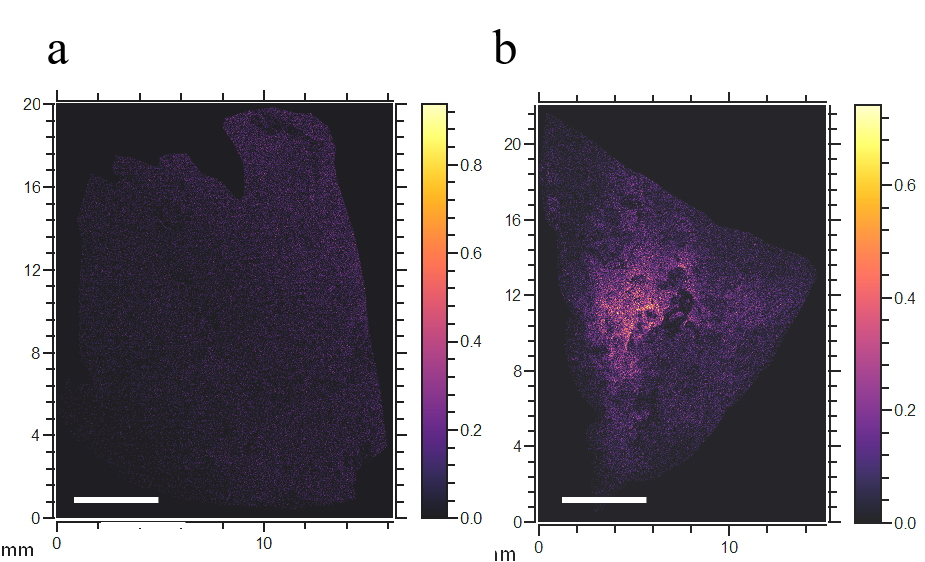


Figure S1 ToF-SIMS ion images for iron (*m/z* 55.9) for (a) healthy human donors and (b) COPD (GOLD IV) patients


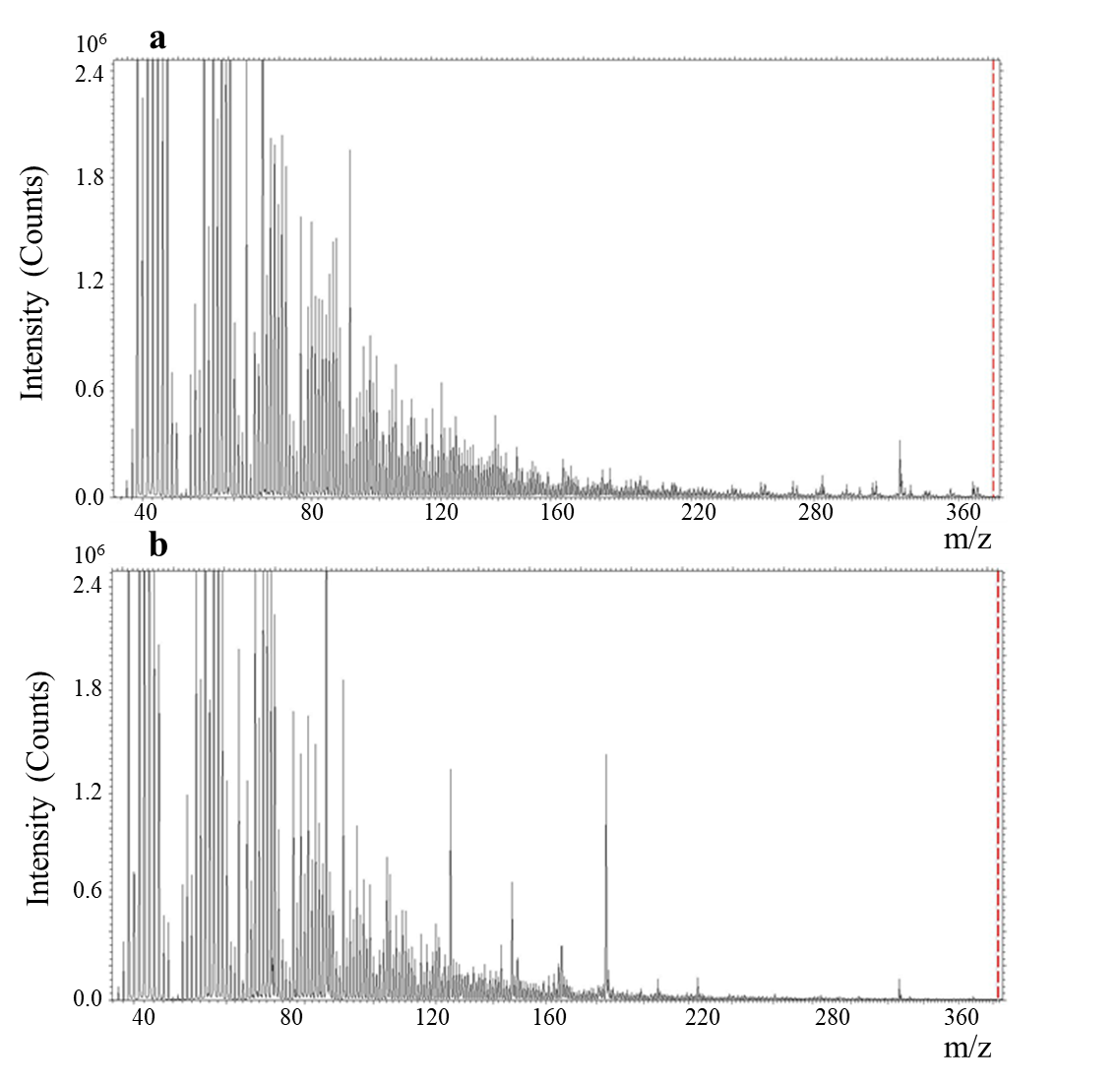


Figure S2 1. Full spectra for the full scan image for both a) healthy human donor and b) COPD.
